# Microform Calibration Uncertainties of Rockwell Diamond Indenters

**DOI:** 10.6028/jres.100.041

**Published:** 1995

**Authors:** J. F. Song, F. F. Rudder, T. V. Vorburger, J. H. Smith

**Affiliations:** National Institute of Standards and Technology, Gaithersburg, MD 20899-0001

**Keywords:** calibration uncertainty, diamond indenter, HRC, microform, Rockwell hardness, traceability

## Abstract

National and international comparisons in Rockwell hardness tests show significant differences. Uncertainties in the geometry of the Rockwell diamond indenters are largely responsible for these differences. By using a stylus instrument, with a series of calibration and check standards, and calibration and uncertainty calculation procedures, we have calibrated the microform geometric parameters of Rockwell diamond indenters. These calibrations are traceable to fundamental standards. The expanded uncertainties (95 % level of confidence) are ±0.3 μm for the least-squares radius; ±0.01° for the cone angle; and ±0.025° for the holder axis alignment calibrations. Under ISO and NIST guidelines for expressing measurement uncertainties, the calibration and uncertainty calculation procedure, error sources, and uncertainty components are described, and the expanded uncertainties are calculated. The instrumentation and calibration procedure also allows the measurement of profile deviation from the least-squares radius and cone flank straightness. The surface roughness and the shape of the spherical tip of the diamond indenter can also be explored and quantified. Our calibration approach makes it possible to quantify the uncertainty, uniformity, and reproducibility of Rockwell diamond indenter microform geometry, as well as to unify the Rockwell hardness standards, through fundamental measurements rather than by performance comparisons.

## 1. Introduction

The Rockwell hardness test is a mechanical testing method for evaluating a property of metal products. Rockwell hardness tests are probably used more than all other hardness tests and other mechanical tests (tension, torsion, creep, etc.) combined [1]. Among Rockwell hardness tests, the Rockwell C test, which employs a diamond indenter, 98 N (10 kgf) preliminary test force and 1471 N (150 kgf) total test force, is the most widely used method. The Rockwell C hardness reading (HRC) is calculated from the net increase of the penetration depth *d*, when the force on the diamond indenter is increased from the preliminary test force to the total test force and then returned to the preliminary test force. The Rockwell C hardness reading is determined by [2–4]:

$$HRC=100-\frac{d}{0.002\;\text{mm}}.$$

Like many physical concepts and physical properties to be quantified, Rockwell hardness tests are different from the measurements of most classical measurable quantities, either base quantities (such as length, time, and mass), or derived quantities (such as velocity and density). The strict physical definitions and functional relationships of these classical measurable quantities are independent of measurement methods. Therefore, different methods and instruments can be used to measure the same quantity independently, to test for systematic biases between techniques, to improve measurement technique, and to reduce measurement uncertainty. We use the term methods divergence [5] to refer to systematic biases between different techniques. Rockwell hardness tests, however, are totally based on experiments, and therefore cannot be quantified without reference to a particular method of measurement [6].

The definition of Rockwell hardness comes from written standards [2–4], which include a detailed description of the measurement method: the geometry of the “indenter”, the construction of the machine by which the indenter is applied, and the way in which the machine is to be operated. There is more than one written standard, so there is more than one scale of Rockwell hardness. Rockwell hardness tests have no unit that is independent of such a measurement method. Rockwell hardness readings cannot be entered into algebraic equations to define other measurable quantities (although they are sometimes used in empirical equations that relate hardness to another property for a category of materials) [7].

Since Rockwell hardness readings are defined by the measurement method described in the written standards [2–4], the “correct” Rockwell hardness readings come from the correctness of the realization of these written standards by using a standardized Rockwell testing machine, diamond indenter, and standardized testing conditions. The uniformity and reproducibility of Rockwell hardness tests come from the uniformity of the testing machines and diamond indenters, as well as the verification methods used to test and to maintain the uniformity and reproducibility of the machines and indenters [6].

According to ISO and ASTM standards [2–4], verification methods include direct and indirect verifications. Direct verification consists of separate verifications of testing force, indenter geometric parameters, and the displacement of the measuring device [2]. Direct verifications are based on fundamental measurements, and are traceable to fundamental standards (force, length, angle, etc.). Direct verification lays a foundation to maintain the uniformity and reproducibility of Rockwell hardness tests. Based on the direct verifications of testing force and displacement of the measuring device, standardized deadweight Rockwell testing machines have been developed in several national laboratories [8,9], with measurement repeatability better than ±0.1 *HRC* [9].

On the other hand, direct verification of the Rockwell diamond indenter microform geometry has been a major uncertainty source in Rockwell hardness tests. Historically, optical projection was the only method for these measurements [1]. During the 1940s and early 1950s, Tolmon and Wood [10] at NPL (National Physical Laboratory, U.K.) developed an apparatus using a rotary stage and optical interferometer to measure cone angle and spherical tip radius of the diamond indenter. Based on this work, in 1978 Nash [11] at NPL developed a new method by combining the optical interferometer with a commercial LVDT transducer to measure the spherical radius of the diamond tip. In 1988 Barbato and Desogus [12] at IMGC (Istituto di Metrologia “G. Colonnetti,” Italy) developed another apparatus with an air bearing on the rotary stage and an inductive displacement transducer with probe contacting the measured diamond indenter for determining the radius. In 1967 Yamamoto and Yano [13,14] at NRLM (National Research Laboratory of Metrology, Japan) developed a micro-collimator by using different numerical apertures to measure the spherical radius and form error of the Rockwell diamond indenter. The expanded uncertainties (95 % level of confidence) by these methods were reported to be of the order of micrometers [11,12,14], or sometimes even larger [11].

These expanded uncertainties are of the same order of magnitude as the tolerance requirement of calibration-grade Rockwell diamond indenters as specified in ISO and ASTM standards (200 μm ± 5 μm) [2,4]. Because a more conclusive inspection of the diamond indenter has been impractical, performance comparisons, or indirect verifications, must therefore be an integral part of the inspection procedure [15]. This comparison is performed by using a standardized deadweight Rockwell hardness machine kept as the national standard, and a standardized diamond indenter, which is recognized as the reference indenter at the national level [4] (there is no international standard realization [7]). Standardized Rockwell hardness testing blocks are used as transfer standards [16] for these performance comparisons to control the uniformity of Rockwell diamond indenters as well as Rockwell hardness tests.

The indirect verifications have been successfully used to keep the uniformity of Rockwell hardness tests in a “closed” quality control loop, i.e., within a company, a country, or even an international calibration network, as long as all of their reference standards are traceable to the same “master” standards, i.e., standardized Rockwell hardness machine and diamond indenter. By this method, three U.S. manufacturers maintained a stated uncertainty^1^ of ±0.3 *HRC* for their company’s products as early as the 1950s [1]. At the same time, another U.S. company, by including the uncertainty of the testing blocks, maintained a stated uncertainty of ±0.5 *HRC* [1]. An international comparison was carried out in 1984 under the auspices of OIML [17]. The majority of the countries possessing national hardness standards took part. Wilson Instruments of Bridgeport, Connecticut, was the American participant, since at that time the United States had no national laboratory for Rockwell hardness standards. The results obtained within the countries of the EEC showed good agreement (±0.3 *HRC* between 30 *HRC* to 65 *HRC*). This was probably because of the international comparison previously carried out within the Bureau Communautaire de Reference of EEC [12,18].

However, when the comparison of Rockwell hardness tests is performed in an “open” quality control loop, which includes several companies or countries with their independently established quality control procedures and defined standard Rockwell hardness machines and diamond indenters, the comparison differences are significant. At NIST, an intercomparison study on Rockwell hardness testing blocks made by seven manufacturers [19] showed that the comparison differences are even greater than the tolerance limits for standardized hardness testing blocks as specified in ISO and ASTM standards [2,4]. For HRC hardness blocks, the differences are between 1 *HRC* and 1.2 *HRC* in the range of 25 *HRC* to 63 *HRC* [19]. For the international comparison mentioned above [17], the comparison for countries not in the EEC showed a maximum variation of ±0.9 *HRC* in the range of 30 *HRC* to 65 *HRC*. This variation is very high compared with the uncertainties considered acceptable in critical industrial applications [12].

With the development of standardized deadweight Rockwell hardness testing machines [8,9], research work has been carried out on the disagreement of Rockwell hardness tests. A general conclusion is that the microform geometry calibration uncertainties of the Rockwell diamond indenter are largely responsible for the differences in Rockwell hardness tests [6, 8, 11–15, 20, 21]. It has been found that, provided the same diamond indenter is used, machines of quite different design are capable of giving the same scales of hardness if attention is given to the uncertainty of every factor affecting the measurements [8]. However, as long as more than one indenter is involved, it is difficult to achieve agreement between two machines over the whole range of hardness values [8]. An incorrectly manufactured diamond indenter exhibits a complex geometric shape, which is difficult to measure accurately. Previous efforts have ignored these complexities due to the difficulty of measurement [22]. Furthermore, because of the large uncertainties in performance comparison tests, not only are “poor indenters” being accepted, but it is possible that “good indenters” are being rejected or relapped because their performance is erroneously thought to be in error [22].

There is a strong industrial requirement to unify Rockwell hardness tests. NIST has addressed this problem by establishing the National Rockwell Hardness Calibration Laboratory. One important step is to establish the microform calibration system for Rockwell diamond indenters. At the NIST surface and microform calibration laboratory, we have established and used a proven calibration procedure [23,24] for surface texture calibrations of our sinusoidal profile roughness specimens [25] and other surface specimens and engineering surfaces. This calibration procedure includes instrument calibration and check calibration, surface measurements and check measurements utilizing a series of calibration and check standards [23–26] and uncertainty calculation procedures [27,28]. Based on this previous work, we are now using a commercial stylus instrument, combined with the use of calibration and check standards, and calibration and uncertainty calculation procedures, for the microform calibration of Rockwell diamond indenters [29,30]. This approach can be easily and independently implemented and is traceable to fundamental standards with acceptably small measurement uncertainties. The instrumentation and calibration procedures also allow for the measurement of profile deviation from the least-squares radius and cone flank straightness. Engineering features of the diamond indenter, such as surface roughness and whether it is flat or sharp with respect to a sphere, can be also explored and quantified from these calibrations [29,30].

According to ISO and NIST guidelines for expressing measurement uncertainties [7,31], we developed our uncertainty calculation procedures and calculated the expanded uncertainties (95 % level of confidence) to be ±0.3 μm for the least-squares radius, ±0.01° for the cone angle, and ±0.025° for the holder axis alignment calibrations. These expanded uncertainties are less than one tenth of the tolerance requirements for calibration-grade Rockwell diamond indenters. In this paper, we describe the instrument setup, calibration and check standards, calibration procedures, error sources and uncertainty components, expanded uncertainty calculations, and calibration results. The approach makes it possible to quantify the uncertainty, uniformity, and reproducibility of Rockwell diamond indenter microform geometry, as well as to unify the Rockwell hardness standards, through fundamental metrology rather than by performance comparisons.

## 2. Instrument Setup, Calibration and Check Standards, and Calibration Procedures

The Rockwell diamond indenter is a diamond cone with 120° of cone angle blended in a truly tangential manner with a spherical tip of 200 μm radius. The microform geometry and calibration requirements according to ISO and ASTM standards [2–4], are shown in Table 1. The working-grade indenters are used for the regular Rockwell hardness tests, while the calibration-grade indenters are reserved for calibrations of standardized hardness blocks.

From the point of view of basic metrology and standardization, we have established the metrology requirements for the microform calibration of Rockwell diamond indenters [6,29,30]. Previous measurement techniques [1,10–14] cannot meet these calibration requirements. Our approach is to use a commercial stylus instrument (Form Talysurf^2^ manufactured by Rank Taylor Hobson, Leicester, England.) A stylus-laser transducer with 2 μm stylus radius and 60 mm arm length is used. The profile sampling interval is 0.25 μm and the profile quantization step is 0.01 μm, both of which are traceable to an optical wavelength.

**Fig. 1:**
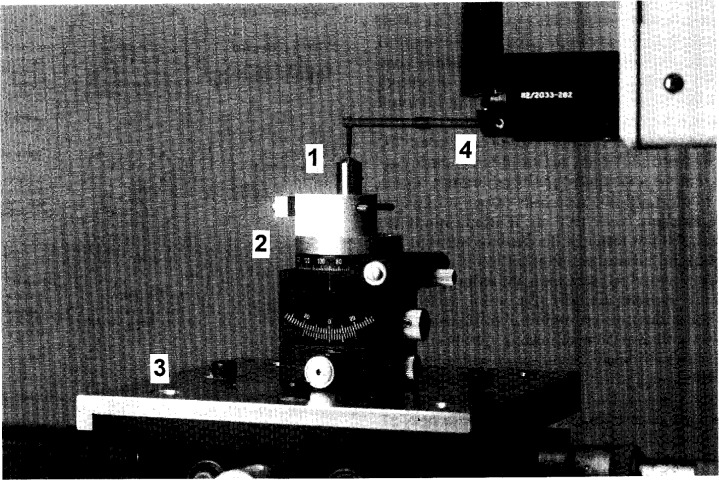
Stylus instrument for the microform calibration of Rockwell diamond indenters: (1) Rockwell diamond indenter; (2) rotary stage; (3) *x*–*y* stage; (4) stylus-laser transducer.

The instrument setup is shown in Fig. 1. The Rockwell diamond indenter (1) is set on a rotary stage (2), which is mounted on an *x*–*y* stage (3). The holder axis and the rotation axis of the rotary stage are previously aligned relative to the instrument’s *z*-axis. The software package of the instrument makes it possible to use different window sizes and either least-squares arc fitting or line fitting for determining the least-squares radius and profile deviation, cone angle and cone flank straightness. The holder axis alignment error can also be calibrated by using the rotary stage and a least-squares sinusoidal data fitting algorithm (see Appendix A). It is also possible to measure the surface roughness by using an analysis option in the instrument’s software. The profile deviations of the spherical tip of the Rockwell diamond indenter can also be explored and quantified [30].

**Fig. 2:**
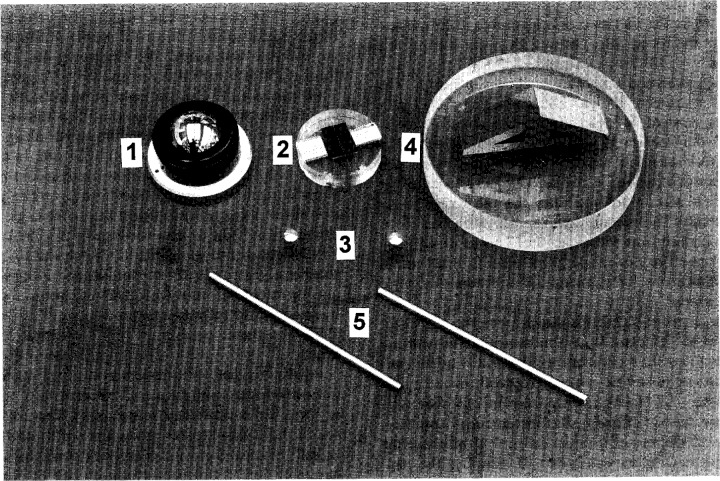
Calibration and check standards: (1) 22 mm radius standard calibration ball; (2) 0.204 mm radius standard wire; (3) 0.199 mm radius ruby balls; (4) 120° angle gauge block; (5) ground steel bars.

Our effort has focused on the traceability, reproducibility, and uncertainty of our calibration procedure. The traceability and uncertainty depend upon the establishment of calibration and check standards, as well as on the calibration procedure. The calibration and check standards are shown in Fig. 2. A 22 mm radius standard ball (1) supplied by the instrument manufacturer is used for the instrument calibration. Concerning the selection of check standards in surface and microform calibrations, one of the important considerations is that the size and form (sometimes even the material) of the check standards should be as close as possible to the measured elements, and with high geometric uniformity, high material stability, and small calibration uncertainty [23,24,29]. Since we do not have a perfect Rockwell diamond indenter to serve as a check standard, we separate these calibrated elements into two categories: 200 μm radius and 120° angle, and use different check standards. For the least-squares radius calibrations, a standard wire (2) and two ruby balls (3) are used as the check standards. Their radii are selected close to the nominal 200 μm radius of the diamond indenter. The ruby balls are mounted on a steel indenter-shaped holder with the spherical tip presented to the stylus just as with the actual diamond indenters. The actual diameters of these check standards are measured interferometrically and are traceable to the wavelength of light. For the 120° cone angle calibrations, a 120° angle gauge block (4) (assembled using two 30° angle gauge blocks wrung on an optical flat) is used as the angle check standard. These angle gauge blocks are also traceable to the NIST angle standard. The 120° angle gauge block is used only at its top area, with a 400 μm measurement length on each side close to the vertical interface. This is the same trace length used for the cone angle calibration of the diamond indenters. The ground steel bars (5) (Fig. 2), with the same diameter as the holder of the Rockwell diamond indenter and with good geometric uniformity, are used for the alignment of holder’s axis to the rotation axis of the rotary stage, as well as to the instrument’s *z*-axis.

**Fig. 3:**
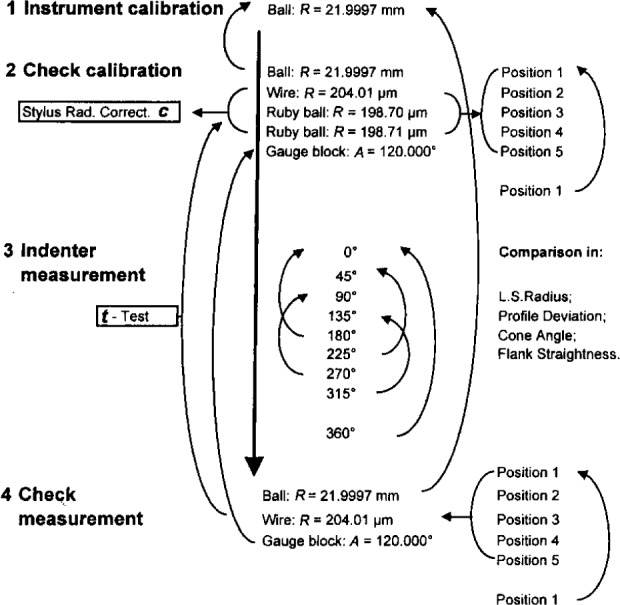
Calibration procedures and measurement assurance loop of Rockwell diamond indenters.

The calibration procedure is shown in Fig. 3. The stylus instrument is first calibrated using the 22 mm radius standard ball, and the instrument calibration is checked by measuring the same standard ball, as well as the check standards: standard wire and ruby balls. Each standard is measured at five sections and remeasured at the first section for checking the measurement repeatability. The average stylus radius correction (*c*) is also obtained from these measurements as we will discuss later. The 120° angle gauge block is measured in several sections for checking the correctness of angle calibrations.

The diamond indenter is then measured in eight sections at 45° intervals. By this measurement sequence, it is easy to test the measurement repeatability by comparing the results between every two opposite measurement positions [29]. However, since there are only four independent measurements in this measurement sequence, the small degrees of freedom (*v* = 4 − 1) increases the *t*-factor value (discussed in Sec. 3), as well as the combined calibration uncertainties. We intend to use a nine-section measurement sequence with 40° intervals to increase the degrees of freedom to *v* = 8, in order to reduce the *t*-factor, as well as the expanded uncertainty.

In each measurement section, by moving the *x*–*y* stage, the stylus is first crowned on the top point of the diamond indenter. Then, a 1.2 mm (±0.6 mm) traced profile with 4800 data points is taken. By windowing the central ± 100 μm range and using least-squares arc fitting, the least-squares radius and profile deviation from the radius is determined. By windowing the left part from −450 μm to −100 μm and the right part from +100 μm to +450 μm, the cone angle and cone flank straightness error are determined with a least-squares straight line fit. The measurement length along each flank of the cone is approximately 404 μm, in accordance with 0.4 mm minimum cone flank measurement length as required by the ISO standards [3,4]. The holder axis alignment error is calculated from cone angle measurements at eight or nine sections by a least-squares sinusoidal data fitting algorithm (see Appendix A). The roughness measurements of the diamond indenter can also be calculated from the windowed profile sections by selecting the software analysis options of the instrument (roughness parameters and appropriate cutoff length).

Finally, another measurement at the 360° section is made to compare with the measurement at the 0° section to check the measurement repeatability. This comparison includes least-squares radius and profile deviation, as well as cone angle and cone flank straightness. We have also done comparisons for each pair of opposite measurement positions, i.e., 0° and 180°, 45° and 225°, 90° and 270°, and 135° and 315°. These comparisons have shown very good measurement repeatability for the 19 diamond indenters we have calibrated to date [29].

The last calibration step is a closure check by remeasuring the 22 mm radius standard ball, standard wire, and angle gauge block. The standard wire is measured at five sections at the same locations as before. A *t*-test is used to test for significant differences between these values and the values measured at the beginning. If no significant difference is found in the diamond indenter calibration loop, all of these calibration data are input to our software package to calculate the calibration uncertainties and to print out the calibration report.

## 3. Error Sources, Uncertainty Components, and Combined Calibration Uncertainties

**Fig. 4:**
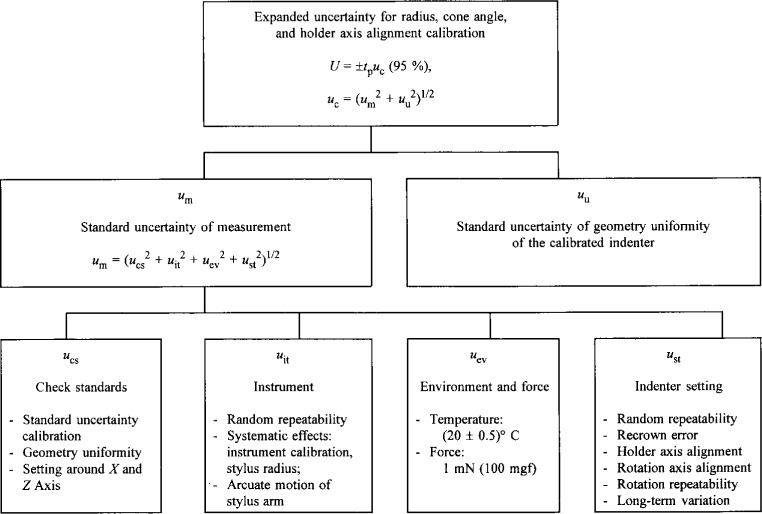
Error sources, uncertainty components, and combined uncertainties of Rockwell diamond indenters.

We developed our uncertainty calculation procedures for the Rockwell diamond indenter calibration (Fig. 4) according to ISO and NIST guidelines for expressing measurement uncertainties [7,31]. The expanded uncertainty *U* with 95 % confidence level for the calibrations of least-squares radius, cone angle and holder axis alignment error is expressed by

$$U=\;\pm \;t_{p}u_{c},$$ (1)

where *t*_p_ is the *t*-factor determined from the confidence level (95 %) and the effective degrees of freedom *v*_eff_ using the Welch-Satterthwaite formula [7,31]

$$v_{\text{eff}}=\frac{u_{c}^{4}(y)}{\sum_{i=1}^{n}c_{i}^{4}u^{4}(x_{i})/v_{i}}\leq \sum_{i=1}^{n}v_{i}.$$ (2)

Where *c_i_* ≡ ∂*f*/∂*x_i_*, *f* is the function that relates the measurand to the input quantities, *u*(*x_i_*) is the *i*th component of standard uncertainty, *v_i_* is the number of degrees of freedom of each component, and *u_c_* is the combined calibration uncertainty which includes the combined standard uncertainty of measurement *u*_m_ and the standard uncertainty from geometric uniformity of the calibrated diamond indenter *u*_u_:

$$u_{c}={\left(u_{m}^{2}+u_{u}^{2}\right)}^{1/2}.$$ (3)

The combined standard uncertainty of measurement *u*_m_ is obtained from various uncertainty components: the standard uncertainties from check standards *u*_cs_, instrument *u*_it_, environment *u*_ev_ and the settlement of the diamond indenter on the rotary stage *u*_st_:

$$u_{m}={\left(u_{\text{cs}}^{2}+u_{\text{it}}^{2}+u_{\text{ev}}^{2}+u_{\text{st}}^{2}\right)}^{1/2}.$$ (4)

The standard uncertainty of the check standards, *u*_cs_, is a combination of standard uncertainties due to the check standard’s calibration uncertainty (standard wire, ruby balls, and angle gauge block), the geometric uniformity of the check standards, and the settlement error of these check standards around the instrument’s *x* and *z* axis. The standard uncertainty of the instrument, *u*_it_, is a combination of standard uncertainties due to the instrument’s random repeatability, systematic effects such as instrument calibration, stylus radius, and arcuate motion of the stylus arm. The standard uncertainty of the environment, *u*_ev_, includes the uncertainties due to the temperature variation and the contact force. The standard uncertainty of the diamond indenter’s settlement, *u*_st_, is a combination of standard uncertainties due to the random variation of the settlement of the diamond indenter on the holder of the rotary stage, the repeatability of recrowning the stylus on the top point of the measured diamond indenter, the rotary stage’s holder and rotation axis alignment error, the rotation repeatability during the calibration process, and the rotation’s long-term variation.

All of these uncertainty components are classified into two categories [7,31], Type A evaluations of standard uncertainties which are evaluated by statistical methods and Type B evaluations of standard uncertainties which are evaluated by other means. For Type A evaluations of uncertainties, independent observations under the same measurement conditions *q*_1_,*q*_2_, ⋯,*q_k_*,·⋯,*q_n_* are obtained, and differ in value because of random variations in the influence quantities. The mean $\bar{q}$, the experimental standard deviation *s*(*q_k_*), the experimental standard deviation of the mean $\left(s\bar{q}\right)$, and degrees of freedom *v* are calculated as [7,31]:

$$\bar{q}=\frac{1}{n}\sum_{k=1}^{n}q_{k}$$ (5a)

$$s^{2}(q_{k})=\frac{1}{n-1}\sum_{k=1}^{n}{\left(q_{k}-\bar{q}\right)}^{2}$$ (5b)

$$s^{2}(\bar{q})=\frac{s^{2}(q_{k})}{n}$$ (5c)

v=n−1 (5d)

For Type B evaluation, the uncertainty is a subjective quantity whose value is to be obtained from experience or from knowledge of the measurement procedure. Depending on the information available, several methods have been suggested for determining Type B standard uncertainties [7,31]. In the microform calibrations of Rockwell diamond indenters, for example, when the standard wire was calibrated at the NIST dimensional calibration laboratory, the expanded uncertainty (which comes from the calibration history of different standard wires in this laboratory) is reported as between 0.025 μm and 0.05 μm, with coverage factor of *k* = 3 and thus a 3 standard deviation estimate. If we quote this expanded uncertainty as (0.0375 ± 0.0125) μm, the standard uncertainty is *u*(*x_i_*) = (0.0125 ± 0.0042) μm. The degrees of freedom can also be calculated by [7,31].

$$v=(1/2){\left[\delta u(x_{i})/u(x_{i})\right]}^{-2},$$ (6)

where δ*u*(*x_i_*)/*u*(*x_i_*) can be considered as the relative uncertainty of the standard uncertainty *u*(*x_i_*):

$$\delta u(x_{i})/u(x_{i})=0.0042/0.0125\approx 1/3.$$ (7)

Therefore, the degrees of freedom is *v* = 4.5 ≈ 4, and the Type B evaluation of standard uncertainty can be expressed as *u*(*x_i_*) = 0.013 μm, *v* = 4 (see Table 2).

When all of these uncertainty components are tested and calculated and their corresponding standard deviations and degrees of freedom are input to our combined calibration uncertainty software, the combined standard uncertainty *u*_c_ and the effective degrees of freedom *v*_eff_ can be calculated from Eqs. (2) and (3), from which the expanded uncertainty *U* (95 % level of confidence) can be calculated using Eq. (1).

## 4. Expanded Measurement Uncertainty for Least-Squares Radius Calibrations

A standard wire and two ruby balls are used as the check standards for the calibration of the least-squares radius of Rockwell diamond indenters. Table 2 shows the expanded uncertainty obtained by using the standard wire as a check standard. The standard wire is calibrated interferometrically with the standard uncertainty of 0.013 μm and degrees of freedom *v* = 4 as mentioned above. The geometric uniformity of the standard wire is statistically tested at five sections located at its middle part at 1 mm spacing. The standard uncertainty of the mean radius is 0.046 μm with degrees of freedom of *v* = 4 (Table 2). The standard uncertainties from rotational errors of the standard wire around the instrument’s *z* and *x* axis are obtained from tests and geometric calculations, and are shown in Table 2.

The standard uncertainties from the environment include the temperature variation in the calibration laboratory, which is no more than (20 ± 0.5) °C, and the error caused by the contact force of 1 mN (100 mgf) between the diamond stylus (*r* = 2 μm) and the standard wire (*R* = 204 μm). Both of these uncertainties can be calculated by using standard formulae [32]. The standard uncertainties and degrees of freedom are shown in Table 2.

An important uncertainty component for the calibration of the least-squares radius of a Rockwell diamond indenter is the stylus radius. The stylus radius is certified by the instrument manufacturer as (2 ± 0.5) μm, and a 2 μm stylus radius is used as one of the calibration constants in the instrument’s software package. However, the actual stylus radius can vary in the range of ±0.5 μm, which will directly introduce a systematic error in the least-squares radius calibration of the diamond indenter. In order to obtain an acceptably small measurement uncertainty, it is important to test the actual stylus radius and include a stylus radius correction *c* in the least-squares radius calibrations of Rockwell diamond indenters. It is also necessary to estimate the standard uncertainty of the methods divergence by using different methods for determining the stylus radius correction *c*, and include this uncertainty into the expanded uncertainty.

There are different methods for testing stylus radius [33]. Three methods have been used to measure the actual stylus radius of our stylus instrument and the methods exhibited reasonable agreement [29]. By the razor blade method [33,34], we measured the average least-squares radius of the stylus with a two-standard deviation uncertainty to be (1.56 ± 0.05) μm. However, the actual contact between the stylus and the surface depends more on the outer profile envelope than on the least-squares radius. Hence, this value represents a lower limit. As a second method, we measured a pair of well-matched convex and concave lenses with the same 12.4710 mm radius. We obtained an average stylus radius value with a two standard deviation uncertainty of (1.52 ± 0.22) μm. For the third method, we measured the standard wire and ruby balls using our stylus instrument. The nominal stylus radius of 2 μm was one of the instrument calibration constants. We compared the measured radii of these check standards with that determined with an optical interferometer at the NIST dimensional calibration laboratory. We obtained an average stylus radius with two standard deviation uncertainties of (1.74 ± 0.05) μm, (1.67 ± 0.11) μm, and (1.71 ± 0.07) μm corresponding to different daily calibrations [29].

We currently use the last method, i.e., by measuring the standard wire and ruby balls, for determining the stylus radius correction value *c*, because it is more precise than the other two methods and it assesses stylus radius under conditions very close to those of the measurement itself. In addition, this approach enables us to combine the instrument calibration check and the determination of the stylus radius correction in the same step of the calibration procedure of a Rockwell diamond indenter (see Fig. 3). Therefore, we do not need to test separately the actual stylus radius before every calibration of a Rockwell diamond indenter. By this method, it is also possible to compensate for potential systematic errors from the stylus instrument’s hardware and software package during the least-squares radius measurements of the Rockwell diamond indenter. Since the actual radius of these check standards is very close to the measured diamond indenters, and the shape of the ruby balls is the same as the measured spherical tip of the indenters, the contact situation between the stylus and the check standards is very close to that of the measured diamond indenters. We have used this principle for selecting check standards in surface metrology [23], as well as in surface microform geometry calibrations [29].

We have also compared the measurement results from the three check standards over several days and we include an uncertainty for this component in the expanded uncertainty (1 standard deviation or 1 S.D. = 0.069 μm as shown in Table 2). If the stylus radius correction *c* is obtained by averaging three stylus radius corrections obtained from three check standards (each measured at five sections), this uncertainty component can be reduced by a factor of $1/\sqrt{3}$ (1 S.D. = 0.040 μm).

Another error comes from the arcuate motion of the stylus transducer. The rotation of the 60 mm stylus arm around the *y* axis results in a measured profile (raw profile) that is deformed by the mixed data in the *x* and *z* coordinates. In order to correct this deformation, the instrument is first calibrated by a 22 mm radius standard ball. An internal (and proprietary) algorithm calculates a series of calibration constants and stores these constants in the instrument’s software. By using these calibration constants, the raw profile is corrected into a “modified” profile from which various geometric parameters are calculated. However, after calibrating the instrument with a 22 mm radius standard ball, we use it to measure a very small radius of 200 μm and a cone angle over a small lateral range: ±100 μm for the radii, ±(100 to 450) μm for the cone angle. The measurement variations should be previously tested at different stylus positions in the *z* direction. The maximum range of the stylus motion in the *z* direction is about ±2.5 mm. We measured the standard wire and the 120° angle gauge block at five different positions: 0 mm, ±1 mm, and ±2 mm. From the maximum variation of these measurements, we estimate the standard deviation and degrees of freedom, and include these values in the expanded uncertainty (see Tables 2, 3, and 4).

Concerning the evaluation of the Type B standard uncertainties and their degrees of freedom, we need information on the distribution probability, the maximum variation range, and the confidence level. This information usually comes from experience with the measurement procedure. For example, since we can easily control the stylus position within ±0.3 mm in the *z* direction during the calibration of Rockwell diamond indenters, we consider the tested value of the maximum measurement variation in a ±2 mm range to have a confidence level of no less than 95 %. Therefore, we can transfer the tested maximum variation (0.31 μm) for the least-squares radius measurements into the standard uncertainty (*s* = 0.077 μm, see Table 2). From our measurement experience, we also estimate that this determination has a reliability no worse than 50 %. This may be taken to mean that the relative uncertainty of the standard uncertainty is δ*u*(*x_i_*)/*u*(*x_i_*) = 0.50 [7]. From Eq. (6), the degrees of freedom is *v_i_* = (0.50)^−2^/2 = 2 (see Table 2).

Another uncertainty source comes from the variation of recrowning the stylus when measuring the diamond indenter from one section to another. The standard uncertainty for the least-squares radius and cone angle measurements is statistically measured and included in Tables 2, 3, and 4, respectively.

When using the standard wire as a check standard for calibration of the least-squares radius, the combined standard uncertainty is *u*_m_ = 0.12 μm with a degrees of freedom of *v*_eff_ = 7 (Table 2). The expanded uncertainty with 95 % confidence level is *U*_m_ = ±*t*_p_*u*_m_ = ±0.29 μm (Table 2). We have also developed a similar measurement uncertainty budget for using the two ruby balls as the check standards. The geometric uniformity of the ruby balls is not as good as that of the standard wire. In addition, there is a recrown uncertainty in the *y* direction when using the balls. Therefore, the expanded uncertainties (95 %) are higher, 0.31 μm and 0.33 μm, respectively. If we use all three check standards (i.e., one standard wire and two ruby balls) to determine the stylus radius correction and control the calibration process, the expanded uncertainty for the least-squares radius calibration can be reduced to ±0.26 μm (95 %).

## 5. Expanded Uncertainty for Cone Angle Calibrations

A similar procedure is used for calculating the expanded uncertainty for the cone angle calibration (see Table 3). The standard calibration uncertainty for the angle gauge block is reported as ±0.25”. Since our check standard is composed of two angle gauge blocks, the standard uncertainty is $\sqrt{2}\times 0.25”\approx 0.35”\approx 0.0001{}^{\circ}$. The standard uncertainty from the angle gauge block’s geometric uniformity is statistically tested as 0.0004°. The standard uncertainties for the rotation of the angle gauge block around the *x* and *z* axis of the stylus instrument, the random repeatability of the instrument, the rotation of the stylus arm, and the recrown repeatability of the stylus on the top point of the measured diamond indenter are also evaluated by using the same method as described in Sec. 4. The standard uncertainties with their degrees of freedom are shown in Table 3.

Another uncertainty component comes from the difference of the measurement positions between the original calibrations of the angle gauge blocks and the measurement of the angle gauge block as the check standard. When calibrating the angle gauge blocks, the measurement positions are located close to the center of their working surfaces. However, when using the angle gauge block as a check standard to check the stylus instrument calibration, the measurement positions are located close to the top edge of the angle gauge blocks in order to be consistent with the measurement conditions of the Rockwell diamond indenter. Because of the form error of the working surfaces of the angle gauge blocks, a measurement uncertainty is involved. We have measured this uncertainty by shifting the positions from the center of the working surfaces to close to the edge of the angle gauge block. From the maximum variation and the estimated confidence level, the standard uncertainty is obtained as 0.0025° (see Table 3). The combined standard uncertainty for calibration of the cone angle of the Rockwell diamond indenter is *u*_m_ = 0.0036° with degrees of freedom of *v*_eff_ = 7 (Table 3). The expanded uncertainty (95 %) is *U*_m_ = ±*t*_p_*u*_m_ = ±0.0085°, less than 1/10 of the tolerance requirement for the calibration-grade Rockwell diamond indenters specified in ISO and ASTM standards (Table 1) [2,4].

## 6. Expanded Uncertainty for Holder Axis Alignment Calibrations

In order to calculate the holder axis alignment uncertainty, we developed a least-squares algorithm for sinusoidal data fitting (see Appendix A). When the cone angle measurements at eight (or nine) sections are used in this fitting procedure, the holder axis alignment error, the phase angle, and the standard uncertainty of the holder axis orientation can be calculated.

**Fig. 5:**
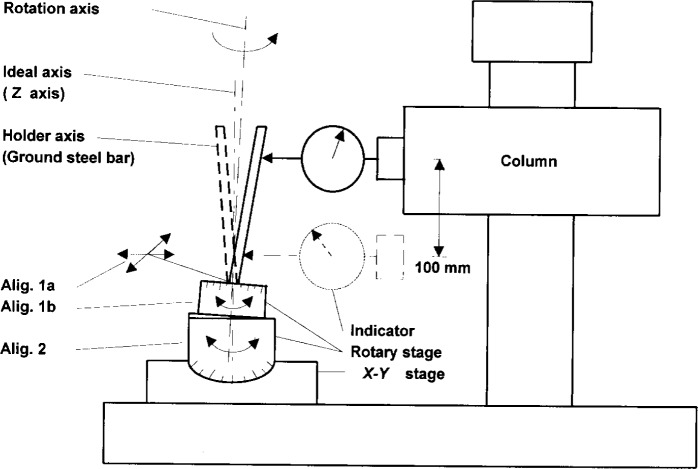
Rotary stage alignment: Alig. 1a and 1b, alignment of holder axis with rotation axis; Alig. 2, alignment of rotation axis with ideal axis (instrument’s *z*-axis).

The expanded uncertainty for holder axis alignment calibration largely depends on the rotary stage alignment. Before the calibration of a Rockwell diamond indenter, the rotary stage should be well aligned by using a ground steel bar and a dial indicator at two adjustment sections (Fig. 5). The lower section is located at the same level as is used for the diamond indenter calibration, while the upper section is taken 100 mm higher. First of all, the holder axis of the rotary stage is aligned as close as possible to the rotation axis by adjustment of the mounting screws and shims (Alig. 1a and 1b). This alignment is verified when the indicator readings exhibit a minimum variation at both sections, when the rotary stage rotates 360° around the *z* axis. After that, the rotary stage is aligned with its rotation axis as close as possible to the ideal axis (i.e., the instrument’s *z* axis). This alignment may be performed by rotating the lower level of the rotary stage around the *x* and *y* axes (Fig. 5, Alig. 2). By moving the indicator along the standard bar from the lower to the upper section, the parallelism with the *z* axis of the instrument is tested until minimum variation is obtained from the indicator’s readings. These tests should be performed in two perpendicular sections (*xz* and *yz*).

**Fig. 6:**
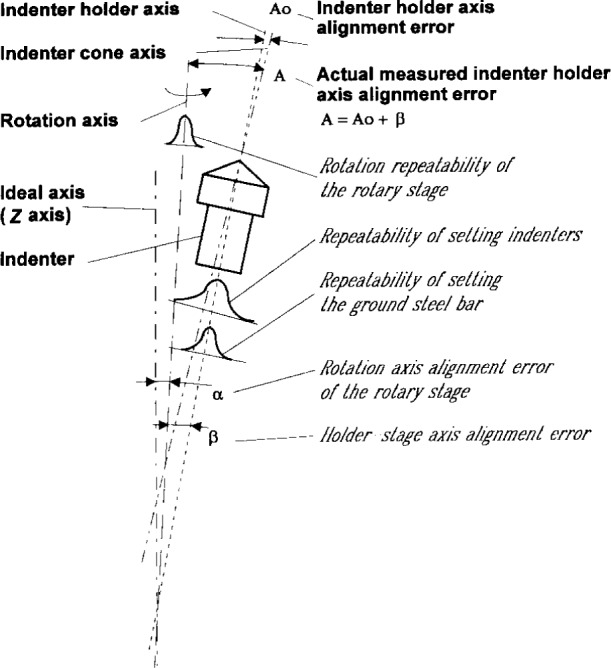
The systematic and random errors from the alignment of the rotary stage.

After completing these alignments, the expanded uncertainty for the holder axis alignment calibrations is determined (Table 4). The systematic errors are mainly from the holder-stage axis alignment error *β* (see Fig. 6). The holder axis alignment error of the Rockwell diamond indenter *A*_o_ is defined as the angle shift between the holder axis and the cone axis (see Fig. 6). However, because of the holder-stage axis alignment error *β*, the actual measured holder axis alignment error of the Rockwell diamond indenter is *A* = (*A*_o_ + *β*). The rotation axis alignment error of the rotary stage *α* (Fig. 6) is derived from the least-squares sinusoidal data fitting as a constant offset (see Appendix A), which has no direct effect on the holder axis alignment calibration of the Rockwell diamond indenters. Another systematic effect comes from the geometry error of the ground steel bar, including its dimension and form error, which is shown in Table 4. The random effects are from the rotation repeatability of the rotary stage, the repeatability when setting the diamond indenter in the holder of the rotary stage for calibrations (see Fig. 1 and Fig. 6), the repeatability when setting the ground steel bar in the holder of the rotary stage for the alignments (see Fig. 5 and Fig. 6), and the long term variation of the rotary stage alignment. All of these systematic and random effects are tested and evaluated. The standard deviations and degrees of freedom are shown in Table 4.

For Type A standard uncertainties, attention should be paid to the difference between the experimental standard deviation *s*(*q_k_*), and the experimental standard deviation of the mean $s(\bar{q})$ [Eqs. (5b) and (5c)]. For example, since there is a mechanical slip fit between the holder on the rotary stage and the Rockwell diamond indenter, as well as the ground steel bar, random variations come from the settlement of the diamond indenter in the holder during the calibration (Fig. 1), as well as from the settlement of the ground steel bar in the holder for the alignment procedure (see Fig. 5). We have determined this standard uncertainty to be *u* = 0.007° (*n* = 10). When we consider the random variation of setting the Rockwell diamond indenter, we take the standard uncertainty as *u*(*x*) = 0.007° (see Table 4 and Fig. 6). However, when we align the axis of the rotary stage, in order to obtain a small alignment error, we test the mean position of the ground steel bar first, and then set the ground steel bar on its mean position for the alignment. Therefore, the standard uncertainty is $u(\bar{x})=u(x)/\sqrt{n}=0.0022{}^{\circ}$ (*n* = 10, see Table 4 and Fig. 6).

The combined standard uncertainty for calibration of the holder axis alignment error is *u*_m_ = 0.011° with effective degrees of freedom *v*_eff_ = 31 (Table 4). The expanded uncertainty is *U*_m_ = ±*t*_p_*u*_m_ = 0.023° (95 %). This is less than 1/10 of the tolerance requirement for calibration-grade diamond indenters (Table 1). This value may also be reduced further by improving the rotary stage alignment.

## 7. Expanded Uncertainties for Profile Deviation Calibrations

The profile deviation calibrations include the local profile deviations from a least-squares radius fit and the cone flank straightness relative to a least-squares mean line fit. To test the expanded uncertainties, we need specimen standards with minimum surface geometric error to simulate the least-squares radius fit and least-squares mean line fit. This is performed by using our ruby balls and 120° angle gauge block check standards. We measured the two ruby balls with 24 measurements (12 measurements each) at various radial sections. From these measurements, we found that the maximum profile peak and valley deviations from the least-squares radius are within the range of ±0.1 μm. This value includes the surface geometric error of the ruby balls, as well as the expanded uncertainty of the profile deviation from the least-squares radius. We therefore infer the expanded uncertainty of the profile deviation from the least-squares radius as less than ±0.1 μm, with a confidence level no less than (1−1/24) or ≈95 %. We also measured 24 sections on the 120° angle gauge block, and obtained the expanded uncertainty for the cone flank straightness calibrations as ±0.05 μm (95 %).

## 8. Calibration Results

We have so far calibrated 19 Rockwell diamond indenters. Eight of them were previously measured at other laboratories. Our calibration results showed very good measurement repeatability [29], as well as significant differences with the previous measurements. Among the eight diamond indenters measured previously at other laboratories, six of them had passed the requirements of working-grade, and two of the six indenters had passed calibration-grade requirements both for geometric measurements and performance tests. However, our calibrations showed that only three of the indenters are qualified as working-grade, and none are qualified as calibration-grade Rockwell diamond indenters [29,30].

One of the measurement comparisons is shown in Table 5. This Rockwell diamond indenter (C 14738) was measured by two other national laboratories. In 1986 it qualified as a working-grade indenter. In 1991, it qualified as a calibration-grade indenter both by geometric measurements and performance tests. This Rockwell diamond indenter was considered to be one of NIST’s master indenters for calibrating standardized Rockwell hardness blocks. Our calibration indicated that this indenter does not qualify as a working-grade Rockwell diamond indenter [29,30]. The most significant differences between laboratories occur for the least-squares radius measurement. The cone angle measurements, however, show good agreement. This finding is consistent with the other seven Rockwell diamond indenter measurement comparisons.

## 9. Conclusion and Suggestions

1. Previous measurement techniques cannot meet the microform calibration requirements of Rockwell diamond indenters. Their expanded uncertainty (95 %) for the least-squares radius is of the same order of magnitude as the tolerance requirement of calibration-grade Rockwell diamond indenters. National and international comparisons of Rockwell hardness tests have shown significant differences. The microform calibration uncertainties of Rockwell diamond indenters are largely responsible for these differences.
2. A stylus instrument, combined with a series of calibration and check standards, and calibration and uncertainty calculation procedures, can be used to calibrate the microform geometry of Rockwell diamond indenters in accordance with the definitions specified in ISO and ASTM standards [2–4]. The microform calibration is traceable to fundamental standards with acceptably small uncertainties. The expanded uncertainties (95 %) are less than one tenth of the tolerance requirements of calibration-grade Rockwell diamond indenters: ±0.3 μm for least-squares radius; ±0.01° for cone angle; and ±0.025° for holder axis alignment calibrations. The profile deviation from the least-squares radius and the cone flank straightness can also be calibrated with acceptably small uncertainty. The surface roughness and the profile deviation of the spherical tip of the Rockwell diamond indenter can also be explored and quantified from these calibrations [30].
3. The combined standard uncertainties *u*_c_ for the least-squares radius, cone angle, and holder axis alignment calibrations come from the combined standard uncertainty of measurement *u*_m_ and the standard uncertainty from the geometric uniformity of the calibrated Rockwell diamond indenter *u*_u_. The combined standard uncertainty of measurement *u*_m_ comes from different standard uncertainty components: the check standards *u*_cs_, stylus instrument *u*_it_, calibration environment *u*_ev_, and the settlement of the diamond indenter in the rotary stage *u*_st_. All of these uncertainties are categorized as Type A or Type B standard uncertainties. Various methods have been described for testing and evaluating these standard uncertainties and the associated degrees of freedom.
4. Before the stylus instrument can be generally used for Rockwell diamond indenter microform calibrations, the measurement reproducibility should be first verified. This verification can be made by an intercomparison among different laboratories, with their independently qualified stylus instruments (or other measurement techniques), calibration and check standards, and calibration and uncertainty calculation procedures, to measure the same Rockwell diamond indenters. The acceptable comparison reproducibility (95 %) is *D* = (*U*_1_^2^ + *U*_2_^2^)^1/2^, where *U*_1_ is the expanded uncertainty (95 %) in Lab 1, while *U*_2_ is the expanded uncertainty (95 %) in Lab 2. For the least-squares radius calibrations, if the measured components have good geometric uniformity, for example, like our standard wire or ruby balls, the comparison reproducibility (95 %) should be in the sub-micrometer range. For a Rockwell diamond indenter exhibiting good geometric uniformity, it is reasonable to expect the interlaboratory comparison reproducibility (95 %) for least-squares radius calibrations to be within the range of 1 μm.
5. By this method, the instrument setup, calibration and check standards, and calibration and uncertainty calculation procedures can be easily and independently established with traceability to fundamental standards. This approach has made it possible to quantify the uncertainty, uniformity, and reproducibility of the Rockwell diamond indenter microform geometry, as well as to unify the Rockwell hardness standards, through fundamental metrology rather than by performance comparisons.

## Figures and Tables

**Fig. 7:**
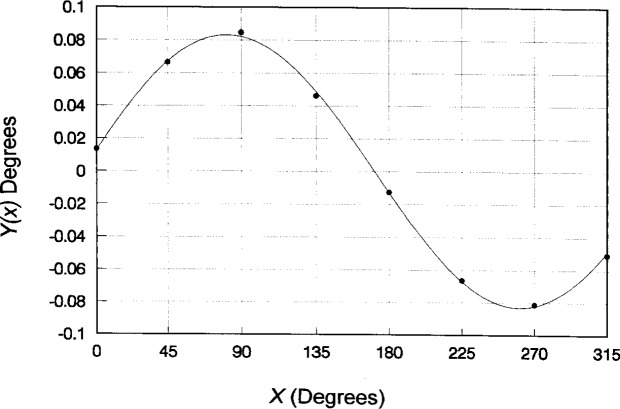
Least-squares sinusoidal data fit for holder axis alignment calibration of Rockwell diamond indenter No. C 14738.

**Table 1:** The microform geometry requirements of Rockwell diamond indenters and NIST expanded uncertainties (95 %)

Microform components and calibration requirements	Working grade		Calibration grade		NIST calibration method and expanded uncertainties (95 %)
	ASTM E18-89a	ISO 716-1986	ASTM E18-89a	ISO 674-1988	
1. Surface finish	The surface of the diamond cone and spherical tip shall be polished for a penetration depth of 0.3 mm and shall blend in a truly tangential manner.				Measurements of R_a_ roughness and profile deviations from the least-squares shapes.
2. Measurement sections	≥4 approx. eq. spaced	≥4	≥8 approx. eq. spaced	≥8 at random	8 sections ×45° or 9 sections ×40°
3. Spherical radius					
3a. Least-squares radius (LSR) (μm)	200±10	200±10	200±5	200±5	±0.3
3b. Max. error of radius (μm)	200±15	200±15	200±7	200±7	±0.3
3c. Profile deviation from the LSR (μm)	±2	±2	±2	±2	±0.1
4. Cone angle					
4a. Mean cone angle	120°±0.35°	120°±0.35°	120°±0.1°	120°±0.1°	±0.01°
4b. Max. error				120°±0.17°	±0.01°
4c. Cone flank straightness (μm)		<1 (at 0.4 mm)		<0.5 (at 0.4 mm)	±0.05
5. Holder axis alignment	±0.5°	±0.5°	±0.3°	±0.3°	±0.025°

**Table 2:** Expanded uncertainty (95 %) for the least-squares radius calibrations of Rockwell diamond indenters using a standard wire as a check standard

Uncertainty component	Source of uncertainty	Type	Standard uncertainty *u*(*x_i_*) (μm)	δ*u*(*x_i_*)/*u*(*x_i_*) for Type B uncertainties	Degrees of freedom *v*
*u*_cs_ Standard uncertainty from the check standard	Standard calibration uncertainty	B	0.013	33 %	4
	Standard wire uniformity	A	0.046		4
	Rotation around *z*-axis	B	0.03	25 %	8
	Rotation around *x*-axis	B	0.0001	25 %	8
*u*_it_Standard uncertainty from the stylus instrument	Random repeatability	A	0.03		9
	Stylus radius correction	A	0.069		2
	Rotation of the stylus arm	B	0.077	50 %	2
*u*_ev_ Standard uncertainty from environment	Temperature, ±0.5° C	B	0.005	25 %	8
	Force, 1 mN (100 mgf)	B	0.01	25 %	8
*u*_st_ Standard uncertainty from the indenter setting	Recrown repeatability of the stylus on the top point of the indenter	A	0.019		8

Combined standard uncertainty: *u*_m_ = 0.124 μm				D.O.F. *v*_eff_ = 7 *t*_p_ = 2.36	

Expanded uncertainty (95 %): *U*_m_ = ±*t*_p_*u*_m_ = 0.293 μm					

**Table 3:** Expanded uncertainty (95 %) for the cone angle calibrations using the 120° angle gauge block as a check standard

Uncertainty component	Source of uncertainty	Type	Standard uncertainty *u*(*x_i_*)	δ*u*(*x_i_*)/*u*(*x_i_*) for Type B uncertainties	Degrees of freedom *v*
*u*_cs_	Standard calibration uncertainty	B	0.0001°	25 %	8
Standard uncertainty from the check standard (120° angle gauge block)	Gauge block uniformity	A	0.0004°		4
	Rotation around *z*-axis	B	0.0012°	25 %	8
	Rotation around *x*-axis	B	(5×10^−5^)°	25 %	8
	Shift of measurement positions	B	0.0025°	50 %	2
*u*_it_	Random repeatability	A	0.001°		8
Standard uncertainty from the stylus instrument	Rotation of the stylus arm	B	0.0017°	50 %	2
*u*_ev_	Temperature, ±0.5° C		0		
Standard uncertainty from environment	Force, 1 mN (100 mgf)		0		
*u*_st_ Standard uncertainty from the indenter setting	Recrown repeatability of the stylus on the top point of the indenter	A	0.011°		8

Combined standard uncertainty: *u_m_* = 0.00358°				D.O.F. *v*_eff_ = 7 *t*_p_ = 2.36	

Expanded uncertainty (95 %): *U_m_ = ±t*_p_*u*_m_ = 0.00848°					

**Table 4:** Expanded uncertainty (95 %) for the holder axis alignment calibrations

Uncertainty component	Source of uncertainty	Type	Standard uncertainty *u*(*x_i_*)	δ*u*(*x*_i_)/*u*(*x*_i_) for Type B uncertainties	Degrees of freedom *v*
*u*_cs_	Geometry error of the bar	B	0.0007°	50 %	2
Standard uncertainty from the ground steel bar	Random repeatability of setting the bar for rotary stage alignment	A	0.0022°		9
*u*_it_	Random repeatability	A	0.001°		8
Standard uncertainty from the stylus instrument	Rotation of the stylus arm	B	0.0016°	50 %	2
*u*_st_	Rotation repeatability	A	0.0003°		9
Standard uncertainty from the rotary stage alignment and indenter setting	Holder-stage axis alignment	B	0.0043°	25 %	8
	Random repeat. of setting indenter	A	0.007°		9
	Recrown repeatability	A	0.0031°		7
	Long-term variation of rotary	B	0.006°	25 %	8

Combined standard uncertainty: *u_m_* = 0.011°				D.O.F. *v*_eff_ = 31 *t*_p_ = 2.04	

Expanded uncertainty (95 %): *U_m_ = ±t*_p_*u*_m_ = 0.023°					

**Table 5:** Comparisons of NIST measurement results with those of two other national calibration laboratories for No. C 14738 indenter

Component	National Laboratory 1 (1986)		Measurement Results National Laboratory 2 (1991)		NIST (1993)	
	Results	Pass/Fail	Results	Pass/Fail	Results	Pass/Fail
1. L.S. radius and profile deviation:						
A. Mean L.S. radius (μm):	210	Pass/Wor.^a^	200+5	Pass/Cal.^b^	213.2^c^	Fail
Expanded calib. uncertainty (95 %)					±2.8	
Comb. meas. uncertainty (1 S.D.)					0.124	
Geometry uniformity (1 S.D.)					0.98	
B. Max. error of radius (μm)	12	Pass/Wor.	+6	Pass/Cal.	15.9	Fail
C. Max. profile deviation (μm)	**±**2	Pass/Cal.	<2	Pass/Cal.	+0.7/−1.1	Pass/Cal.
2. Cone angle and flank straights:						
A. Mean cone angle	120°	Pass/Cal.	120°	Pass/Cal.	120.00°	Pass/Cal.
Expanded calib. uncertainty (95 %)					±0.017°	
Comb. meas. uncertainty (1 S.D.)					0.004°	
Geometry uniformity (1 S.D.)					0.006°	
B. Max. cone flank straights (μm)	0.25	Pass/Cal.			0.27	Pass/Cal.
3. Holder axis alignment error:						
Least-squares mean			0.2°	Pass/Cal.	0.08°	Pass/Cal.
Combined calib. uncertainty (95 %)					±0.023°	
Comb. meas. uncertainty (1 S.D.)					0.011°	
Geometry uniformity (1 S.D.)					0.002°	
4. Special features on surface finish:						
A. Surface roughness: *R*_a_ = (μm)					0.0049	
1 S.D. = (μm)					0.0018	
B. Spherical tip shape					Flat by 0.13 μm from least-squares radius	

a Pass/Wor. denotes passes working grade requirements.

b Pass/Cal. denotes passes calibration grade requirements.

c In October 1994, a fourth national calibration laboratory reported a mean radius of 212 μm.

**Table 6:** Least-squares sinusoidal data fitting for holder axis alignment calibration of Rockwell diamond indenter No. C 14738^a^

*i*	*x_i_*	*Y_i_*	*Y_i_*sin *x_i_*	*Y_i_* cos *x_i_*	*y*(*x*)	*ϵ* =
0	0**°**	0.14495°	0°	0.14495°	0.14434°	0.00061°
1	45°	0.19800°	0.14001°	0.14001°	0.19863°	−0.00063°
2	90°	0.21610°	0.21610°	0.00000°	0.21358°	0.00252°
3	135°	0.17775°	0.12569°	−0.12569°	0.18042°	−0.00267°
4	180°	0.11885°	0.00000°	−0.11885°	0.11858°	0.00027°
5	225°	0.06505°	−0.04600°	−0.04600°	0.06429°	0.00076°
6	270°	0.05025°	−0.05025°	0.00000°	0.04935°	0.00090°
7	315°	0.08075°	−0.05710°	0.05710°	0.08251°	−0.00176°

Sum		1.05170°	0.32845°	0.05152°	Σ*ϵ* = −3.6E-06	
					Σ*ϵ*^2^ = 1.88E-05	

a The results of least-squares sinusoidal data fitting are: *α* = 0.13146°; *A* = 0.08312°; *ψ* = 8.91476°; *s* = 0.00194°.

## Footnotes

1 It is not possible to convert these stated uncertainties to the standard or expanded uncertainties now required by NIST policy.

2 Certain commercial equipment, instruments, or materials are identified in this paper to foster understanding. Such identification does not imply recommendation or endorsement by the National Institute of Standards and Technology, nor does it imply that the materials or equipment identified are necessarily the best available for the purpose.

## 10. Appendix A. Least-Squares Data Fitting For Sinusoidal Functions

### 10.1 Least-Squares Equations

We derive linear equations for the least-squares data fitting of sinusoidal functions of the form

y(x)=α+Asin(x+ψ). (8)

These equations are used for calculations of the offset constant *α*, the amplitude *A*, the phase angle *ψ*, as well as the standard deviation *s* for the sinusoidal data observations *Y_i_*, *x_i_*.

The motivation for this analysis is the estimation of the holder axis alignment error during the microform calibration of Rockwell diamond indenters. The holder axis alignment error *A*_o_ is defined as the angle shift between the holder axis and the cone axis of the Rockwell diamond indenter (see Fig. 6). These measurements are performed by using a rotary stage (see Fig. 1). Because of the holder-stage axis alignment error *β*, the actual measured holder axis alignment error for the Rockwell diamond indenter is *A* = (*A*_o_ + *β*) (see Fig. 6). We have discussed this effect in Sec. 6. At the beginning, the holder axis and the rotation axis of the rotary stage have been well-aligned and tested (see Fig. 5), and those alignment errors have also been included in the combined calibration uncertainty procedure (see Fig. 6 and Table 4). We now introduce the algorithm for least-squares sinusoidal data fitting which is developed for calculating the alignment error *A* and the phase angle *ψ* for the calibrated Rockwell diamond indenter. Meanwhile, the rotation axis alignment error of the rotary stage *α* (as an offset constant) and the standard deviation *s* for the estimated holder axis alignment error *A* can be also derived.

The measurements of Rockwell diamond indenters are performed at *N* positions evenly distributed around the axis of the Rockwell diamond indenter (usually *N* = 8 or 9). From these measurements, we have *N* observations *Y_i_* for the holder axis alignment error corresponding to different angular positions *x_i_* of the calibrated Rockwell diamond indenter. For any angular position *x_i_*, the observed residual error between the measured value *Y_i_* and the least-squares estimated value *y*(*x_i_*) is

$$\epsilon_{i}\equiv Y_{i}-y(x_{i})=(Y_{i}-\alpha )-A\sin(x_{i}+\psi )$$ (9a)

or

$$\epsilon_{i}=(Y_{i}-\alpha )-A\cos\psi \sin x_{i}-A\sin\psi \cos\;x_{i}.$$ (9b)

The total mean square error for the data set (*x_i_*, *Y_i_*) of *N* points is obtained by squaring either Eq. (9a) or (9b) and summing over all points in the data set. Doing this, we obtain the expression for the total mean square error, ***ϵ***_T_^2^ = Σ***ϵ****_i_*^2^:

$$\begin{array}{c} \epsilon_{T}^{2}=\Sigma {\left(Y_{i}-\alpha \right)}^{2}-2A\cos\psi \Sigma (Y_{i}-\alpha )\sin x_{i}\\ -2A\sin\psi \Sigma (Y_{i}-\alpha )\cos x_{i}+A^{2}\cos^{2}\psi \Sigma \sin^{2}x_{i}\\ +2A^{2}\cos\psi \sin\psi \Sigma \sin x_{i}\cos x_{i}+A^{2}\sin^{2}\psi \Sigma \cos^{2}x_{i}. \end{array}$$ (10)

At this point in the development we note that to minimize the total mean squares error ***ϵ***_T_^2^, the parameters *α*, *A*, and *ψ* are selected in the way that

$$\frac{\partial \epsilon_{T}^{2}}{\partial \alpha }=0;\;\frac{\partial \epsilon_{T}^{2}}{\partial A}=0;\;\frac{\partial \epsilon_{T}^{2}}{\partial \psi }=0.$$ (11)

Doing this and noting that Σ*α* = *Nα* one obtains three equations that are non-linear in the unknown parameters *α*, *A*, and *ψ*. The results are:

$$N\alpha +(\Sigma \sin x_{i})A\cos\psi +(\Sigma \cos x_{i})A\sin\psi =\Sigma Y_{i},$$ (12a)

$$\begin{array}{c} {}[(\Sigma \sin x_{i})cos\psi +(\Sigma \cos x_{i})\sin\psi ]\alpha +(\Sigma \sin^{2}x_{i})Acos^{2}\psi \\ +2(\Sigma \sin x_{i}\cos x_{i})A\sin\psi \cos\psi +(\Sigma \cos^{2}x_{i})A\sin^{2}\psi \\ =(\Sigma Y_{i}\sin x_{i})cos\psi +(\Sigma Y_{i}\cos x_{i})\sin\psi , \end{array}$$ (12b)

$$\begin{array}{l} {}[(\Sigma \cos x_{i})\cos\psi -(\Sigma \sin x_{i})\sin\psi ]\alpha \\ +(\Sigma \cos^{2}x_{i}-\Sigma \sin^{2}x_{i})A\sin\psi cos\psi \\ +(\Sigma \sin x_{i}cosx_{i})A(\cos^{2}\psi -\sin^{2}\psi )\\ =(\Sigma Y_{i}\cos x_{i})cos\psi -(\Sigma Y_{i}\sin x_{i})\sin\psi . \end{array}$$ (12c)

Values of the parameters *α*, *A*, and *ψ* that satisfy Eqs. (12a) through (12c) provide a minimum to the total mean square error, ***ϵ***_T_^2^ of the data fit.

### 10.2 Simplification of Non-Linear Least-Squares Equations

Equations (12a), (12b), and (12c) are non-linear in the unknown parameters *α*, *A*, and *ψ*. We now utilize the periodic characteristics of the functions in Eqs. (12) and some useful trigonometric identities to simplify these equations. We note that if *N* is an integer greater than 2, and if the data are sampled at sections *x_i_* = 2π*i*/*N* for *i* = 0,1, ⋯,*N*-1, then the following trigonometric identities hold for the summations:

$$\Sigma \sin x_{i}=0;\;\Sigma \cos x_{i}=0;\;\Sigma \sin x_{i}\cos x_{i}=0.$$ (13a)

$$\Sigma \sin^{2}x_{i}=N/2;\;\Sigma \cos^{2}x_{i}=N/2.$$ (13b)

One may find these identities in many mathematics tables. Reference [35] is a useful source.

Doing this, one obtains the simplified form of Eqs. (12) as:

$$N\alpha =\Sigma Y_{i},$$ (14a)

$$\begin{array}{c} (N/2)A\cos^{2}\psi +(N/2)A\sin^{2}\psi =(N/2)A\\ =[\Sigma Y_{i}\text{sin}(2\pi i/N)]\cos\psi +[\Sigma Y_{i}\cos(2\pi i/N)]\sin\psi , \end{array}$$ (14b)

$$[\Sigma Y_{i}\cos(2\pi i/N)]\cos\psi -[\Sigma Y_{i}\sin(2\pi i/N)]\sin\psi =0,$$ (14c)

where the data are measured at sections *x_i_* = 2π*i*/*N* and all summations are taken over the sequence *i* = 0,1,2,⋯,*N*-1. This sequence assumes *x*_o_ = 0 for the data set.

### 10.3 Parameter Estimates

By requiring the data to be sampled at sections with the angular positions determined by *x_i_* = 2π*i*/*N* and taking *N* as an integer greater than 2, the non-linear Eqs. (12a), (12b), and (12c) are transformed into the simplified linear Eqs. (14a), (14b), and (14c), respectively. These simplified linear equations maybe solved immediately for the unknown parameters *α*, *A*, and *ψ*. The results are:

$$\alpha =(1/N)\Sigma Y_{i},$$ (15a)

A=2R/N, (15b)

$$\tan\psi =[\Sigma Y_{i}cos(2\pi i/N)]/[\Sigma Y_{i}sin(2\pi i/N)],$$ (15c)

$$\cos\psi =[\Sigma Y_{i}sin(2\pi i/N)]/R,$$ (15d)

$$\sin\psi =[\Sigma Y_{i}\cos(2\pi i/N)]/R,$$ (15e)

where Σ is a summation over the sequence *i* = 0,1,2,⋯,*N*-1 and

$$R^{2}\equiv {\left[\Sigma Y_{i}\cos(2\pi i/N)\right]}^{2}+{\left[\Sigma Y_{i}\sin(2\pi i/N)\right]}^{2}.$$ (15f)

We include Eqs. (15c) to (15f) here so that one may unambiguously estimate the phase angle parameter *ψ*.

### 10.4 Standard Deviation of the Estimated Amplitude

The estimated standard deviation *s* of the estimated amplitude *A* can be calculated by

$$s={\left(\frac{\epsilon_{T}^{2}}{n-m}\right)}^{1/2},$$ (16)

where ***ϵ***_T_^2^ = Σ***ϵ***_i_^2^ is the total mean square error, see Eq. (10). (*n*−*m*) is the degrees of freedom. For the holder axis alignment calibration of Rockwell diamond indenters, *n* = *N*, *m* = 3. This standard deviation mainly comes from the geometric uniformity of the calibrated Rockwell diamond indenters, which has been included as an uncertainty component in the combined standard uncertainty of Rockwell diamond indenters (see Fig. 4).

### 10.5 An Example of Least-Squares Sinusoidal Data Fitting

We include here an example of the holder axis alignment calibration of Rockwell diamond indenter No. C 14738. This Rockwell diamond indenter was calibrated at eight sections from 0° to 315°, 45° apart. The measurement data are shown in Table 6, where *x_i_* shows the measurement angular positions, *Y_i_* shows the measurement results of the holder axis alignment error, *y*(*x*) shows the least-squares fitted results, and ***ϵ*** shows the residual error. By using Eq. (15), we calculate the holder axis alignment error as *A* = 0.083° and the phase angle as *ψ* = 8.91°. The offset constant *α* = 0.13° represents the rotation axis alignment error of the rotary stage (see Fig. 6). Therefore, the least-squares fit (shown in Fig. 7) for holder axis alignment of Rockwell diamond indenter No. C 14738 is given by

y(x)=α+Asin(x+ψ)=0.13°+0.083°sin(x+8.91°). (17)

The standard deviation of the least-squares fit is calculated from Eq. (16) as *s* = 0.0019°, which has been included as an uncertainty component in the combined standard uncertainty of this Rockwell diamond indenter.
